# Olfactory Bulbectomy Leads to the Development of Epilepsy in Mice

**DOI:** 10.1371/journal.pone.0138178

**Published:** 2015-09-14

**Authors:** Yifei Jiang, Raymund Y. K. Pun, Katrina Peariso, Katherine D. Holland, Qingquan Lian, Steve C. Danzer

**Affiliations:** 1 Department of Anesthesia, the Second Affiliated Hospital of Wenzhou Medical University, Wenzhou, Zhejiang, China; 2 Department of Anesthesia, Cincinnati Children’s Hospital Medical Center, Cincinnati, OH, United States of America; 3 Division of Neurocritical Care, University of Cincinnati Medical Center, Cincinnati, OH, United States of America; 4 Department of Neurology, Cincinnati Children’s Hospital Medical Center, Cincinnati, OH, United States of America; 5 Departments of Anesthesia and Pediatrics, University of Cincinnati, Cincinnati, OH, United States of America; University of California, Riverside, UNITED STATES

## Abstract

There is a clear link between epilepsy and depression. Clinical data demonstrate a 30–35% lifetime prevalence of depression in patients with epilepsy, and patients diagnosed with depression have a three to sevenfold higher risk of developing epilepsy. Traditional epilepsy models partially replicate the clinical observations, with the demonstration of depressive traits in epileptic animals. Studies assessing pro-epileptogenic changes in models of depression, however, are more limited. Here, we examined whether a traditional rodent depression model—bilateral olfactory bulbectomy—predisposes the animals towards the development of epilepsy. Past studies have demonstrated increased neuronal excitability after bulbectomy, but continuous seizure monitoring had not been conducted. For the present study, we monitored control and bulbectomized animals by video-EEG 24/7 for approximately two weeks following the surgery to determine whether they develop spontaneous seizures. All seven bulbectomized mice exhibited seizures during the monitoring period. Seizures began about one week after surgery, and occurred in clusters with severity increasing over the monitoring period. These results suggest that olfactory bulbectomy could be a useful model of TBI-induced epilepsy, with advantages of relatively rapid seizure onset and a high number of individuals developing the disease. The model may also be useful for investigating the mechanisms underlying the bidirectional relationship between epilepsy and depression.

## Introduction

The most common co-morbidity observed in patients with epilepsy is depression. Studies have reported up to 35% lifetime prevalence rates of depression in patients with epilepsy. Interestingly, the relationship between epilepsy and depression is bidirectional. Patients diagnosed with primary depressive disorder with no history of seizures have an increased risk of developing epilepsy in their lifetime [1–3]. Despite recent interest, the mechanistic relationship between epilepsy and depression remains uncertain [4,5].

In rats, depression has been modeled for several decades using a bilateral olfactory bulbectomy procedure (OBX). OBX has long been known to produce a variety of behavioral changes [6]. Obvious effects include anosmia. Animals also exhibit behavioral changes, however, reminiscent of major depressive disorder [7]. These changes include hyperactivity, increased aggression, reduced sexual activity, altered responses to food rewards and learning deficits [7]. A small number of studies in the literature indicate that rats develop increased neuronal excitability after OBX [8,9], suggestive of pro-epileptogenic changes.

The OBX model was initially developed in rats, but more recently has been extended to mice with generally similar findings [10]; although differences among strains are evident [11]. Like rats, mice exhibit a variety of behavioral changes, including hyperactivity [12] and increased immobility in the forced swim test [13]. Also consistent with rat studies, depressive-like behaviors in mice can be reversed by chronic treatment with antidepressants medications, including amitriptyline [14], citalopram [14] and fluoxetine [15]. Acute antidepressant treatment (<2 weeks) produces no improvement in the OBX model, paralleling clinical practice, in which several weeks of treatment are necessary for efficacy. This excellent predictive validity of the OBX model for known antidepressant drugs justifies its widespread use for depression research [7,10]. Lastly, while OBX isn’t a known cause of depression in humans, traumatic brain injury—which can lead to depression—is commonly associated with damage to the olfactory system[16]. Furthermore, anosmia is increased in patients with major depression [17]. While these clinical associations do not conclusively establish a mechanistic connection, they suggest that OBX in rodents could model some pathogenic aspects of human disease.

For the present study, we queried whether OBX mice reflect the association between depression and epilepsy seen in clinical populations. To assess epileptogenesis in OBX mice, we conducted 24/7 video-EEG monitoring to determine whether the animals develop spontaneous recurrent seizures.

## Materials and Methods

### Animals

All procedures conformed to National Institute of Health guidelines for the care and use of animals. Studies were approved by the Cincinnati Children's Hospital Research Foundation Institutional Animal Care and Use Committee (IACUC) under protocol #IACUC2013-0064. A total of 19 C57Bl/6 mice (Charles River Laboratories International, Inc.), aged between 6–12 weeks (18–21 grams weight) were used in the present study. Twelve of these mice received bulbectomy (OBX) surgery. The OBX procedure produced some mortality, with four of 12 mice dying within one week of the surgical procedure. Data from these animals was not included in the study. One OBX animal that became moribund after nine days was also excluded (this animal did have a seizure on the last day of recording). The final study group of seven mice included five males and two females. In addition, three of the seven mice contained “lox P” sites in exon 5 of the phosphatase and tensin homologue (PTEN) gene as part of another study [18]. These animals did not contain a cre recombinase-expressing transgene to mediate recombination at the lox P sites. Thus, these animals are functionally wildtype, and do not differ phenotypically from the four “true” wildtype C57BL/6 mice. Seven age-matched C57Bl/6 mice (5 male, 2 female) were used as controls. Three of these mice contained lox P sites flanking exon 5 of the PTEN gene, while the remaining four were wildtype animals. No control mice died in the days after electrode implantation surgery, although one mouse became moribund after nine days. Mice were housed on a 14/10 (light/dark) cycle, which is standard practice in the CCHMC vivarium to optimize breeding [19]. Animals had access to food and water *ad libitum*.

### Bulbectomy and electrode implantation

Animals were anesthetized with 3.5% isoflurane in oxygen and then maintained at 0.8–1.5% isoflurane throughout the procedure. Bilateral olfactory bulbectomy and EEG electrode implantation surgeries were performed together. For continuous 24/7 cortical EEG monitoring, animals were implanted with two-lead wireless transmitters (TA11ETA-F10, Data Sciences International, MN). Transmitter leads were placed into two 1 mm diameter holes drilled through the skull overlying the left and right cortical hemispheres (1.5 mm anterior to the lambda suture and 1.5 mm lateral to the midline). Care was taken not to perforate the dura so that skull encapsulation remained intact. The transmitter was inserted into a subcutaneous pocket behind the neck. OBX surgery was performed by drilling two holes through the cribriform plate approximately 1.5 mm anterior to the rostral dorsal cerebral vein (AP +6–7 mm), and 2 mm lateral to the midline. The bulbs were aspirated with a sterilized, blunt ended, 22-guage needle connected to a vacuum line. After removal of the bulbs the cavity was filled with sterile hemostatic sponge (Gelfoam, Pfizer, U.S.A.) as conducted by previous investigators [8,12,13,20–22]. Following the EEG electrode implantation and bulb removal the incision was closed entirely. Animals were administered pain medication (Ibuprofin, 40mg/Kg) orally after recovery from anesthesia. Animals were then placed in individual recording chambers to begin EEG monitoring. Control animals did not undergo OBX surgery.

### 24–7 Video-EEG monitoring

**Fig 1:**
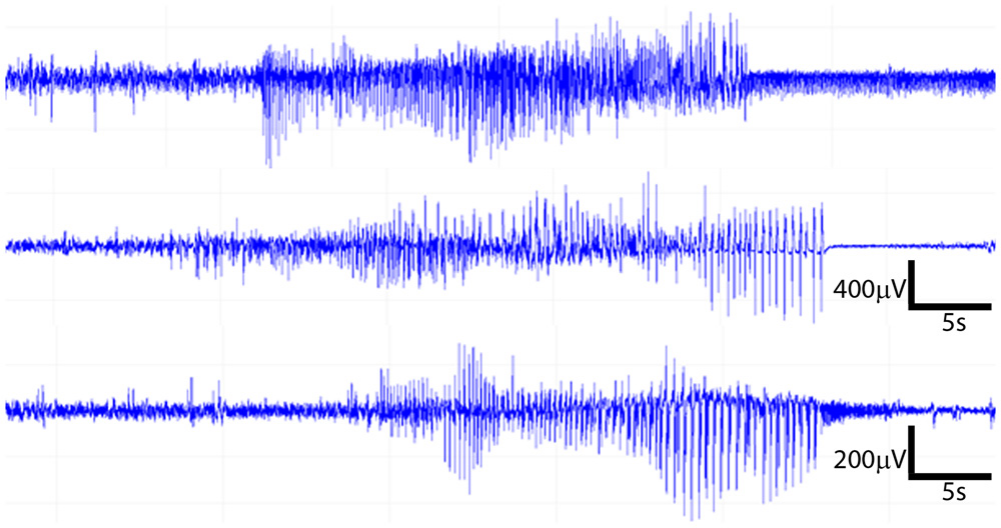
Examples of seizures recorded from three different OBX mice. Scale bars are microvolts (μV) / seconds (s).

EEG and video recordings were performed in an isolated, sterile room equipped with a DSI wireless telemetry system running DATAQUEST A.R.T. (version 4.2) acquisition software (Data Sciences International). The room has the same 14/10 light/dark cycle as the vivarium. The EEG signal was digitized at 1000 Hz and analyzed using Neuroscore software (version 2.1, DSI) by an experimenter unaware of treatment group (OBX vs. control). Seizures were defined as a sudden onset of high amplitude (>2× background) activity with signal progression (a change in amplitude and frequency over the course of the event) and a duration greater than ten seconds (Fig 1).

Behavioral manifestations of seizures were quantified using a modified Racine scale[23] with six stages: 1) immobilization or freezing, 2) mouth movements, facial movements or head bobbing 3) unilateral forelimb clonus, 4) rearing/bilateral forelimb clonus, 5) rearing and falling with bilateral forelimb clonus and 6) running fit. If an animal progressed through multiple seizures stages, the highest score reached was given for the event. An average behavioral score was calculated for each animal [24].

### Perfusion and histological analyses

Following the completion of video/EEG recording, mice were anesthetized with pentobarbital (65 mg/Kg), perfused transcardially with 0.1M ice-cold phosphate buffered saline (PBS) with 1U/ml heparin for 30 seconds, followed by 2.5% paraformaldehyde plus 4% sucrose solution in PBS at room temperature for five minutes. Brains were removed, post-fixed in the same fixative overnight, cryoprotected in an ascending sucrose series (8–10 hours in 10% sucrose in PBS, overnight in 20% sucrose in PBS and one day in 30% sucrose in PBS) and snap-frozen in 2-methyl-pentane cooled to -25°C. Brains were stored at -80°C until sectioning. Sagittal sections were cut on a cryostat at 60 μm. Brain sections were mounted to gelatin-coated slides and slides were stored at –80°C until use.

Sections were stained with the fluorescent Nissl stain NeuroTrace 530/615 (Invitrogen, Grand Island). Sections were used to confirm effective removal of the olfactory bulbs and reveal the extent of any cortical damage produced by the surgery. To quantify the extent of cortical damage, every fourth section through the medial-lateral extent of the brain (L 1.26mm- R 1.26mm; 15 sections/animal from a total of about 60 sections/animal) was imaged using an Olympus BX51 epiflourescent microscope equipped with a 4X, 0.13 NA objective. Sufficient sections were imaged to cover the breadth of the entire damaged region. Images were imported into Neurolucida software (version 5.65; Microbrightfield Inc., Williston, VT) and the damaged brain area from each section was measured. For each brain section, the corresponding plate in Paxinos and Franklin’s mouse brain atlas [25] was identified and anatomical features were used to estimate the volume of cortex lost. The total volume of cortical loss per animal was obtained by multiplying the measured area by the thickness of the tissue section (0.060 mm) examined plus the three unmeasured adjacent sections (0.060 mm + 0.180 mm = 0.240 mm), generating the following equation: volume lost = ([mm^2^ lost in section 1 X 0.240 mm] + [mm^2^ lost in section 5 X 0.240 mm] +…[mm^2^ lost in section 57 X 0.240 mm]. The missing volume from each OBX mouse was used to determine whether there was any correlation between the extent of cortical damage and seizure activity.

### Figure preparation

Images presented in the figures were prepared using Olympus DP controller (version 3.1.1.267). Images were captured from each section with 1/8.0 second exposure time and a 1360*1024 image size. Images were montaged using Adobe Photoshop CS2 (version 9.0.2). Brightness and contrast were adjusted to improve image clarity for publication. In all cases, identical adjustments were made to images meant for comparison.

### Statistics

All statistical tests were performed using Sigma Plot (version 12.3). Parametric and non-parametric tests were used as appropriate depending on normality and equal variance. Specific tests used are noted in the text. Statistical significance was accepted for P values <0.05. Values shown are either means ± standard error or medians [range]. Seizure frequency was calculated by dividing the number of seizures observed by the number of recording days after the first seizure (the latent period was excluded). No differences in seizure frequency (p = 0.20, t-test), duration (p = 0.97, t-test) or behavior score (p = 0.86, Mann-Whitney rank sum test) were found between male and female OBX mice, so data were pooled for analysis. Nonetheless, “n” was low for females (2 mice), so the absence of significant differences should be interpreted cautiously.

## Results

Seven OBX and seven control mice were monitored for spontaneous seizure occurrence. Animals were monitored for approximately two weeks, except for one control which was monitored for four weeks, and one control that became moribund and was euthanized after nine days. Monitoring produced 101 days of 24/7 video-EEG data from the seven OBX animals (range 12–16 days/mouse), and 108 days of data from the seven control animals (range 9–33 days/mouse). No seizures were observed in any control animals.

### OBX mice develop epilepsy

All seven OBX mice experienced spontaneous seizures within 13 days of the procedure, with the earliest seizure manifesting five days after surgery. Examples of recorded seizures are shown in Fig 1. The mean latency to the first spontaneous seizure among the seven epileptic mice was 8.3±1.0 days. EEG seizures were similar to those reported for animals in established models of epilepsy (Fig 1). Notably, EEG recording was begun as soon as the animals recovered from surgery, and early seizures (occurring <24 hours after the lesion) or status epilepticus were not observed in any animals.

EEG seizures were typically accompanied by behavioral changes. In the most severe cases, animals exhibited generalized convulsive seizures, with bilateral forelimb clonus, rearing and falling (Racine scale class 5). One animal exhibited running fits (class 6). Other behavioral changes included freezing, facial/mouth movements, and unilateral and bilateral forelimb clonus. The mean behavioral seizure score for the nine epileptic OBX animals was 3.4 ± 0.4.

**Fig 2:**
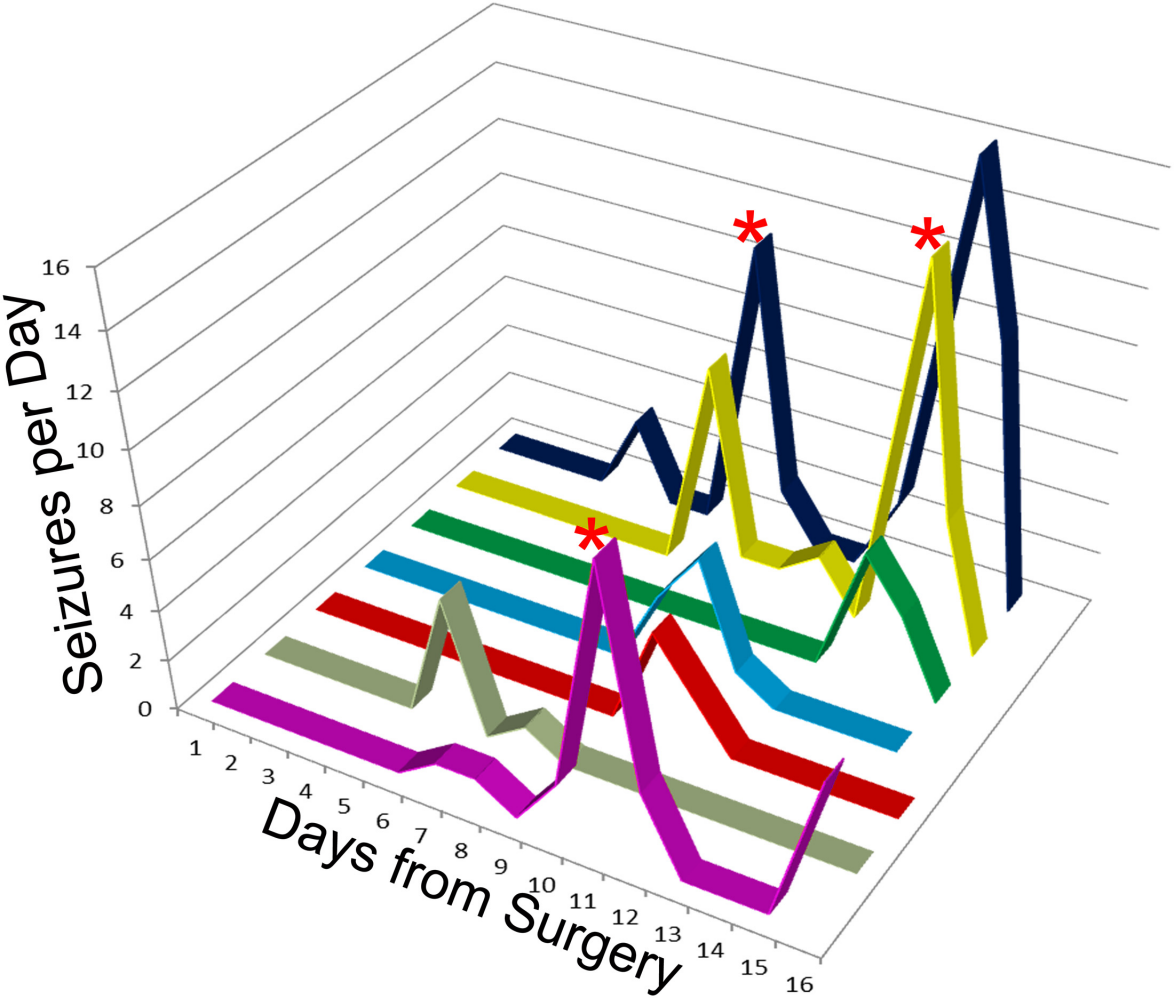
Incidence of seizure activity for the seven OBX animals. Lines show peak seizure number on a given day for each animal. Note that the seizures occurred in clusters rather than being randomly distributed throughout the monitoring period. Three animals had a second seizure cluster (marked by asterisks), data from which were used for Fig 3. No seizures were observed in control animals (not shown).

**Fig 3:**
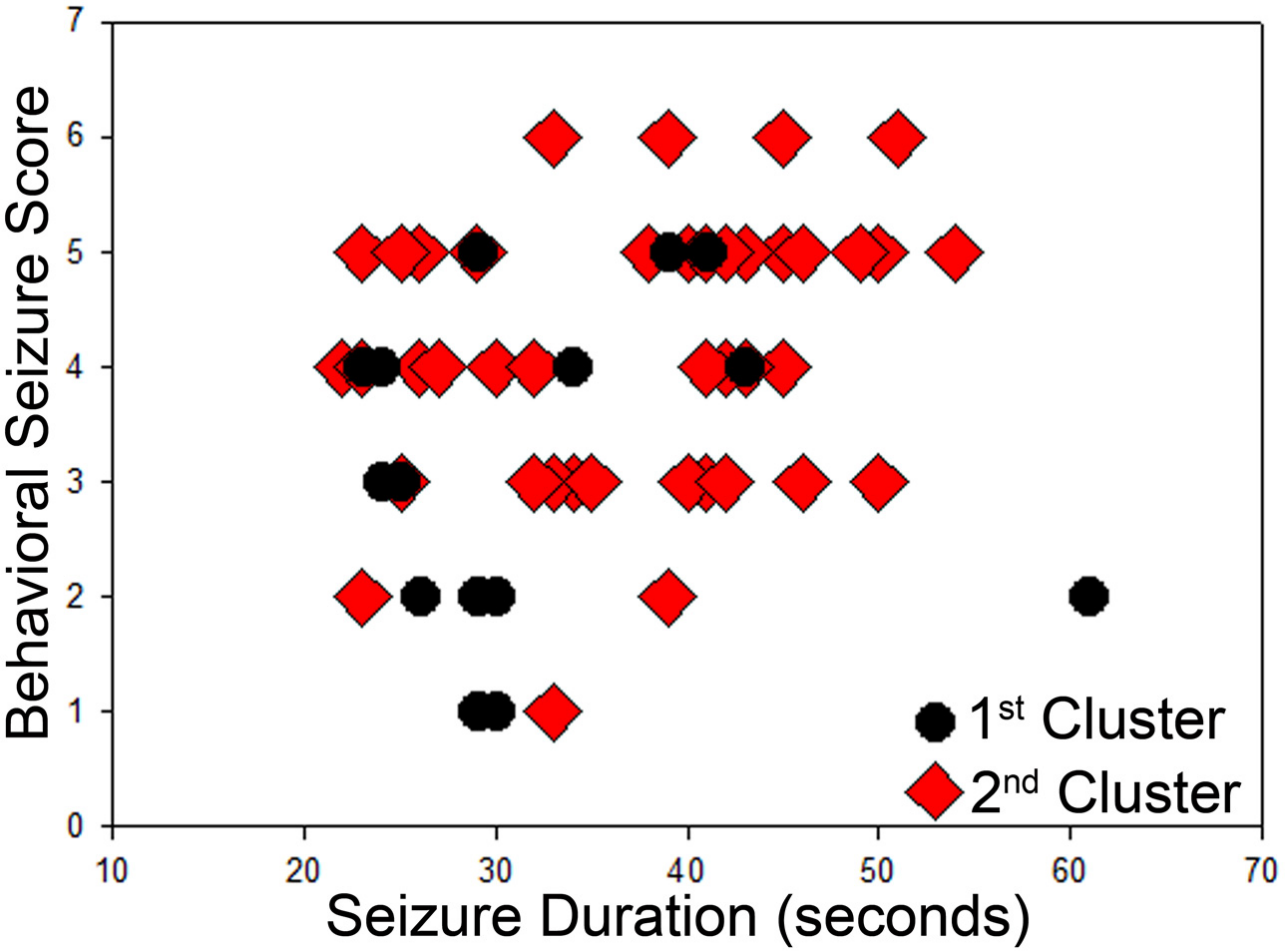
Scatter plot correlating behavioral seizure severity with seizure duration for individual seizures which occurred in either the first (black circles) or second (red diamonds) seizure cluster. Data are from the three animals that had two recorded seizure clusters (see Fig 2). Note the right and upward shift between seizures in the first and second clusters, indicating that seizures in the second cluster were more severe and lasted longer (P<0.001 for both parameters).

The frequency of seizure activity was highly variable among animals, ranging from a maximum of 8.18 seizures/day to a minimum of 0.63 seizures/day. Mean seizure frequency for all eight epileptic mice combined was 3.92±1.21 seizures/day. Seizure incidence for each animal is shown graphically in Fig 2. Seizures occurred in clusters, defined here as the occurrence of two or more seizures in 24 hour period, with at least a 24 hour seizure-free period between clusters. Eleven clusters were identified from the seven mice. Mean seizure occurrence per cluster was 11.1±2.4 (range 2–29). Mean cluster duration was 2.2±0.3 days (range 1–4), with 1.8±0.4 days (range 1–3) between clusters. Seizure clustering is characteristic of many rodent epilepsy models [26,27]and occurs in humans as well [28]. In three animals, two seizure clusters were observed, allowing us to compare seizure duration and severity between the first and second clusters (Fig 3). Among these animals, 17 1^st^ cluster and 48 2^nd^ cluster seizures were quantified. Seizures occurring in the 2^nd^ cluster lasted significantly longer (1^st^, 32.2±2.3 s; 2^nd^, 37.6±1.3 s; P = 0.034, t-test) and were associated with more severe behavioral manifestations (1^st^, 3 (1–5); 2^nd^, 5 (1–6); P<0.001, Mann-Whitney Rank Sum Test). These finding suggest that epilepsy in these animals becomes progressively more severe over time, at least during the first couple weeks.

### The development and severity of epilepsy is not correlated with lesion size

**Fig 4:**
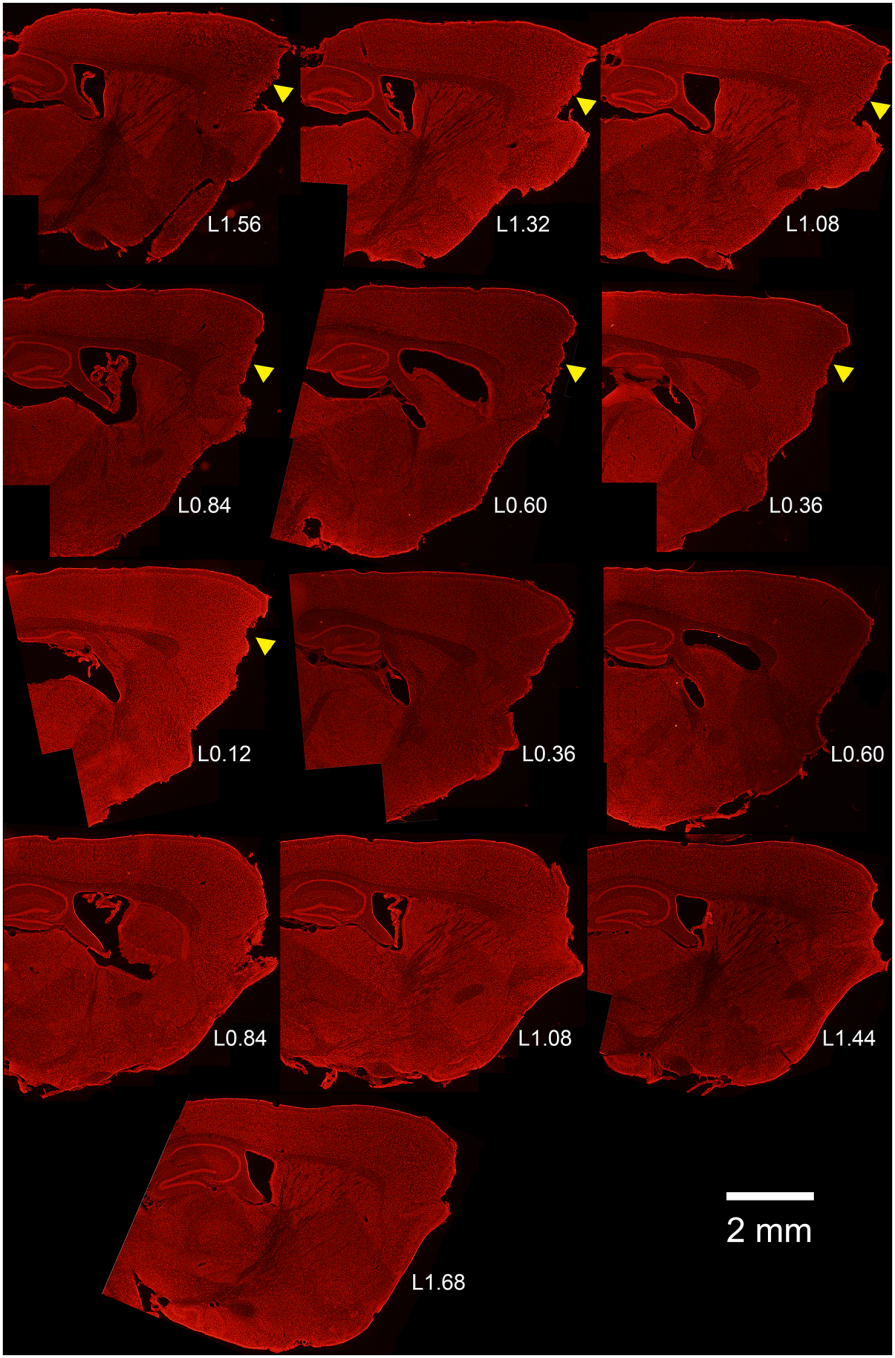
Serial micrographs of Nissl stained sections cut on the sagittal plane through the medial lateral extent through the brain of an epileptic mouse following OBX surgery. Numbers (LX.XX) are distance from midline in mm. Note the effective removal of the olfactory bulb, and modest damage to the frontal lobe in one hemisphere (yellow arrowheads). This pattern and extent of damage is representative of the animals in the study. Scale bar = 2 mm.

**Fig 5:**
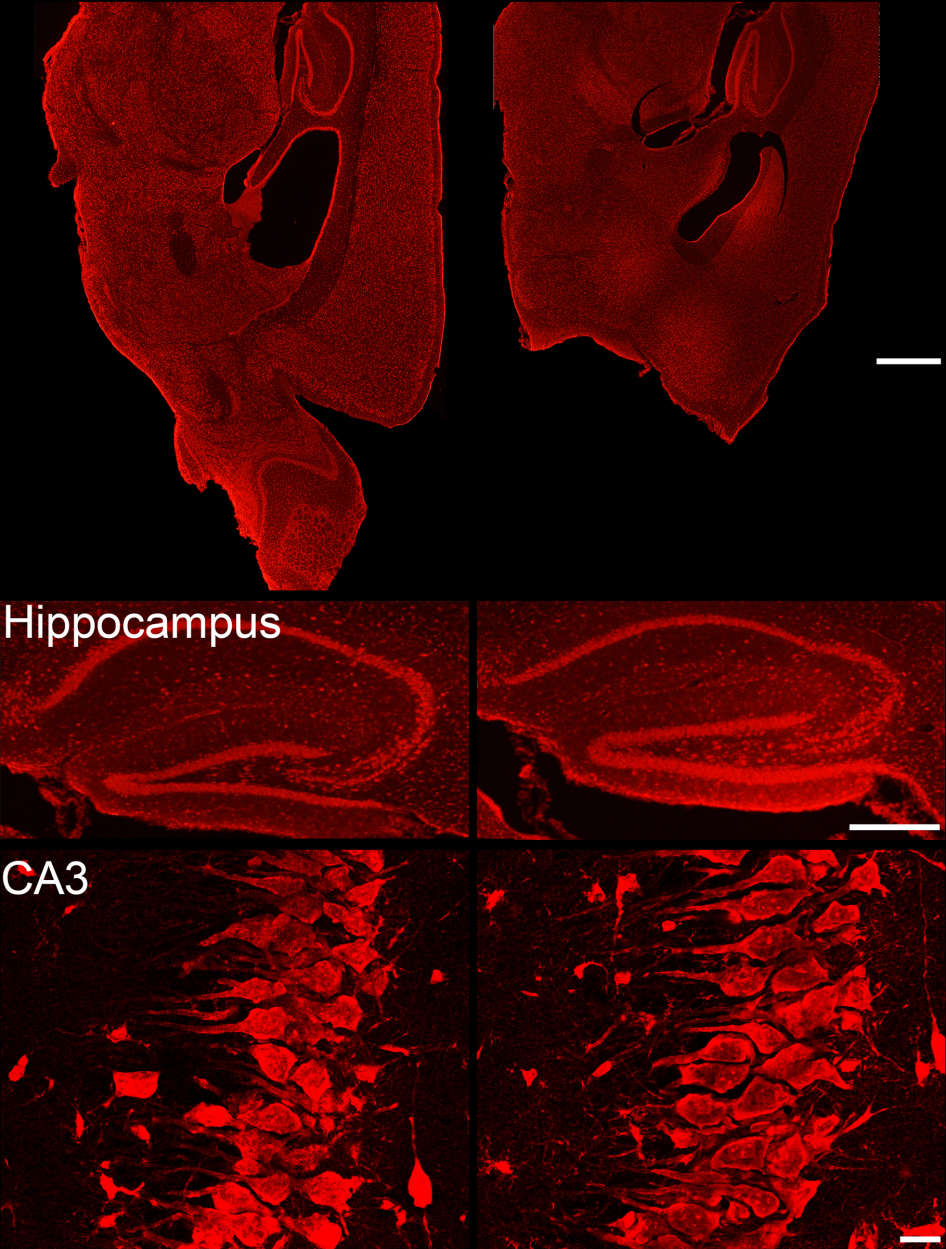
Photomicrographs of Nissl stained sagittal sections from a control mouse (left) and an epileptic mouse with a clean removal of the olfactory bulb. Sections are approximately 0.60 mm lateral to the midline. **Lower panels:** Photomicrographs from control (left) and epileptic (right) mice showing hippocampus and the CA3 pyramidal cell layer. No overt cell loss, disruption or distortion of the overall structure of the hippocampus was evident in hippocampus of any of the animals. Scale bars: Top, 2 mm; middle, 1 mm; bottom, 25 μm.

**Fig 6:**
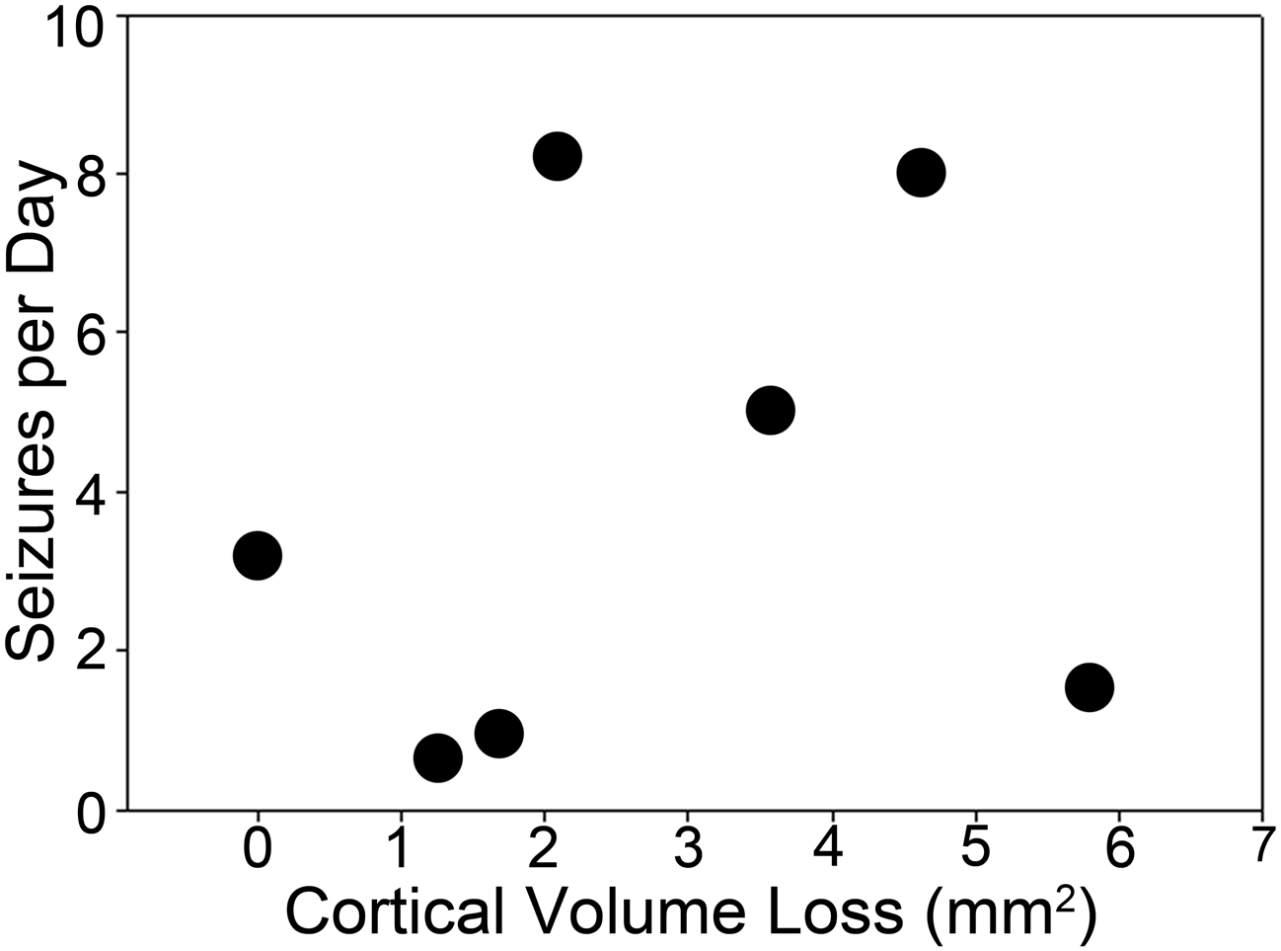
Scatter plot showing the relationship between seizure frequency and estimated cortical tissue loss in mm^3^. A significant correlation between the two parameters was not observed (Pearson product moment, R = -0.218, p = 0.64).

OBX surgery can produce some cortical damage, raising the possibility that seizures might be a consequence of this cortical damage rather than bulbectomy *per se*. To explore this possibility, brain sections from each animal were Nissl stained and the extent of any damage to frontal cortex was quantified. Damage was absent to modest in the animals (Figs 4 and 5). The estimated total volume of cortical tissue lost in the eight animals in the study, excluding the bulb, was 1.36 ± 0.52 mm^3^ (n = 7; range 0.0 to 5.80 mm^3^). For context, an average mouse brain is about 500 mm^3^ [29]. The damage was most evident and extensive in the medial portions of the frontal lobe and became milder towards the lateral edges. No overt cell loss, disruption or distortion of the overall structure of the hippocampus was evident (Fig 5). There was no correlation between the extent of cortical damage and latency to the first seizure (R = 0.415, p = 0.35; Pearson product moment), nor was there any correlation between damage and seizure frequency (R = -0.218, p = 0.64; Pearson product moment, Fig 6). There was also no significant correlation between lesion size and mean seizure duration (R = -0.259, p = 0.57; Pearson product moment). Finally, behavioral scores in each animal were not significantly correlated with the degree of cortical damage (R = 0.252, p = 0.55; Spearman rank order correlation).

## Discussion

We monitored EEG activity in seven OBX mice for about two weeks following surgery. All seven mice exhibited spontaneous seizures during the monitoring period. Spontaneous seizures began five to 13 days after surgery and appeared in clusters, with seizure-free intervals of 1–3 days. Seizures in the second cluster were longer, with more severe behavioral manifestations, indicating that epileptogenesis (as defined by Pitkänen et al) [30] is ongoing over the first few weeks. Together, these findings indicate that OBX surgery, at least as conducted here, can lead to epilepsy in mice. The rapid seizure onset and high percentage of animals affected may make this a useful model for studying epileptogenesis. Furthermore, although behavioral studies were not included here, OBX has been used extensively to model depression in rodents—including studies using similar methodology and the same mouse strain as used here [10–12,14]. The OBX model, therefore, may be ideal for research examining comorbid epilepsy and depression.

### Bulbectomy has been associated with increased excitability

While overt seizures have not been described in previous studies of OBX mice, changes consistent with increased excitability have been observed. Watanabe and colleagues [31], for example, showed that OBX rats have low voltage, fast wave spikes and spindle bursts in cortex and amygdala which persist for up to three months after the procedure. Spontaneous seizures were not observed in this study; however, EEG activity was only monitored for short durations, so occasional seizures would be difficult to detect. Also consistent with a pro-epileptogenic effect of OBX, Nakanishi and colleagues [32] reported increased *in vitro* excitability in amygdala slices prepared from mice that received OBX ten days earlier. Stimulation of the medial amygdala produced burst discharges in slices from OBX rats, but not controls. Furthermore, two studies have demonstrated increased excitability following OBX in the amygdala kindling model of epilepsy [8,9]. In this model, repeated electrical stimulation of the amygdala at constant intensity is used to evoke seizures of increasing severity. Epileptic animals and animals with increased brain excitability kindle “faster,” meaning that it takes fewer electrical stimulations to evoke generalized convulsive seizures. The faster kindling observed in these prior studies would be consistent with the seizure-phenotype described here. Finally, we note that in two earlier studies, OBX was found to *reduce* sensitivity to both electrical and drug-induced (pentetrazol, picrotoxin, aminohexan, strychnine, pilocarpine) seizures in mice [33,34]. These discordant findings might reflect differential mechanisms underlying acutely evoked seizures vs kindling epileptogenesis, and could be an intriguing topic for future investigations. Nonetheless, the majority of prior work supports the conclusion that OBX is pro-epileptogenic.

### Seizures in OBX mice reflect epilepsy

Seizures frequently occur in the hours and days after head injury in human clinical populations. These seizures are not necessarily epileptic, as they often do not persist beyond the acute period, and are presumed to be provoked by transient changes which can affect brain excitability, such as inflammation or swelling. These “early” seizures are defined clinically as occurring during the first week after an injury, and often resolve; while seizures occurring after the first week are indicative of epileptogenesis [35]. In the context of the present study, therefore, it is important to note that four of the seven OBX animals had their first seizure in the second week, and that seizures continued to occur till the end of the recording period in most animals. Although future studies will be needed to ascertain whether seizures persist indefinitely, the present findings strongly suggest that seizures induced by OBX are epileptic, rather than provoked. Consistent with this idea, OBX mice showed clear seizure “clustering”. A subset of animals even went on to have second and third seizure clusters during the recording period (Fig 2). This pattern of clustering is a well-described feature of traditional epilepsy models, such as the pilocarpine and kainic acid-induced status epilepticus models of epilepsy [24,26,27,36]. While the mechanisms underlying clustering remain unknown, the similarity in patterns lends credence to the idea that seizures in the OBX model reflect underlying epilepsy. Indeed, the increased severity of seizures in the second cluster further supports this conclusion (Fig 3).

### The exact nature of the lesion may be critical for epilepsy in this model

A wide range of traumatic brain injuries can produce epilepsy in humans [35] and numerous rodent models of injury-induced epilepsy have now been developed [30]; albeit none that feature damage to the olfactory bulb. The surgical approach used in the present study removed the main olfactory bulbs and removed or damaged the anterior olfactory nuclei, endopiriform nuclei and piriform cortex. It has long been known that olfactory and piriform cortices are highly susceptible to kindling epileptogenesis and other pro-convulsant stimuli [37,38]. There is a strong precedent, therefore, for the conclusion that brain regions damaged by OBX are involved in epilepsy.

OBX also leads to retrograde and anterograde degeneration of neurons in the amygdala and entorhinal cortex in the days and weeks after surgery [39]. Structural changes in hippocampus and entorhinal cortex neurons have also been described [21]. The delay between OBX surgery and the first seizure would provide sufficient time for some of these secondary changes to occur, suggesting that they also may be important for epileptogenesis in this model.

Finally, in the present study, small portions of frontal cortex were damaged in some, but not all animals. The extent to which this is an unavoidable feature of the OBX model is not clear, as detailed histological studies of the lesion are typically not reported in the literature. To facilitate reproducing the model as applied in our hands, however, and to query whether injury severity predicts epilepsy risk, we carefully quantified cortical damage in all seven OBX mice. We did not find a correlation between the degree of brain damage as measured by the volume of missing cortex and the number of seizures; nor was there a correlation between the damaged volume and the duration of seizures. Indeed, one animal with frequent seizures had no overt cortical damage, suggesting that this is not required for epileptogenesis. Nonetheless, we cannot exclude the possibility that damage to frontal cortex is important; or that subtle, surgery-induced injury to this region—not evident in gross histological sections—drives epileptogenesis. Pre-transecting the olfactory tract prior to suction removal of the bulb is reported to minimize damage to the frontal lobe [21] and could prove a useful approach to address this issue in future studies.

### Olfactory bulbectomy as a model of co-morbid epilepsy and depression

Depression is the most common type of psychiatric comorbidity in epilepsy [2]. Correspondingly, most frequently used animal models of epilepsy also feature behavioral symptoms consistent with anxiety/depressive disorders [40–42]. Until now, however, the only animal model of depression that had been tested and demonstrated to have a seizure propensity was the Swim Lo-Active (SwLo) rats. These animals were selectively bred to show increased immobility on the forced swim test, and concomitantly developed a range of other depression-like traits. Epps and colleagues [43] recently demonstrated that these animals have heightened seizure susceptibility. The present demonstration that well-characterized olfactory bulbectomy model of depression also produces epilepsy parallels the strong clinical association between these disorders. With further development, the OBX model could prove to be a useful system to elucidate the mechanistic underpinnings of comorbid epilepsy and depression.
